# Dietary Patterns and Metabolic and Hormonal Parameters in Women with Suspected PCOS

**DOI:** 10.3390/jcm14082708

**Published:** 2025-04-15

**Authors:** Karolina Kowalczyk, Sabina Kadłubek, Aleksandra Krużel, Dominik Sikora, Jakub Daniluk, Paweł Madej

**Affiliations:** 1 Department of Gynecological Endocrinology, School of Medicine in Katowice, Medical University of Silesia, 40-752 Katowice, Poland; 2 Student Scientific Society at the Department of Gynecological Endocrinology, School of Medicine in Katowice, Medical University of Silesia, 40-752 Katowice, Poland

**Keywords:** polycystic ovary syndrome, diet, nutrition, biochemical parameters, metabolic parameters

## Abstract

**Background:** Insulin resistance, visceral adiposity, excess body weight, and symptoms of hyperandrogenism often accompanies Polycystic Ovary Syndrome (PCOS). A balanced diet plays a key role in improving the metabolic and biochemical parameters in affected women. This study aims to assess whether dietary improvements in patients with suspected PCOS may affect the severity of the disease and the metabolic and hormonal profile. **Methods:** The analysis of the relationships between self-declared nutritional changes and biochemical and metabolic parameters included 154 women at the same stage of PCOS diagnosis. **Results:** Over half of participants reported dietary modifications. Women reducing sweets, fatty red meat, and alcohol intake for >6 months had significantly lower total testosterone (TT) levels compared to those who did not (*p* < 0.05). Mean TT levels were: 0.375 ± 0.18 ng/mL (median 0.340) vs. 0.787 ± 2.19 ng/mL (median 0.390) for red meat (*p* = 0.008), 0.359 ± 0.18 ng/mL (median 0.335) vs. 0.681 ± 1.9 ng/mL (median 0.4) for sweets (*p* = 0.02), and 0.388 ± 0.19 ng/mL (median 0.34) vs. 0.917 ± 2.65 ng/mL (median 0.425) for alcohol (*p* = 0.004). Patients with dietary changes in the past 6 months had higher androgen levels, BMI, systolic blood pressure and triglycerides than patients with long-term dietary changes (*p* < 0.05). There were no statistically significant differences in key metabolic and biochemical parameters when comparing self-reported diets based on glycemic index (low vs. high). **Conclusions:** A healthy, balanced diet for women with PCOS requires a multifaceted approach with clear, defined goals. This leads to better results than broad, general dietary recommendations. Long-term dietary changes improve biochemical and metabolic parameters, but maintaining these benefits requires continuous patient motivation.

## 1. Introduction

Polycystic Ovary Syndrome (PCOS) is a prevalent endocrine disorder affecting approximately 10% of women of reproductive age globally [1,2,3,4,5]. The diagnosis of PCOS is based on the presence of at least two out of three criteria: oligo-anovulation, androgen excess (clinical or biochemical), and abnormal ovarian morphology on ultrasound. Irregular menstrual cycles, defined as cycles shorter than 21 days or longer than 45 days within 3 years of menarche, or longer than 35 days after 3 years, are a key indicator. Primary amenorrhea is diagnosed when menstruation has not started by age 15 or 3+ years after breast development. Hyperandrogenism is assessed through biochemical markers, such as free testosterone, or through clinical signs, including acne, female pattern hair loss, and hirsutism. It’s essential for healthcare professionals to recognize the psychosocial impacts of clinical hyperandrogenism and treat concerns about excess hair growth or hair loss seriously, regardless of perceived severity. The modified Ferriman Gallwey score (mFG) should be used to assess hirsutism, considering ethnicity and the potential interference of self-treatment with clinical assessment. A detailed description of the scoring system and the areas assessed can be found in the Section 3.2. It is important to note that while the severity of hirsutism can vary by ethnicity, its prevalence remains similar across different groups [6]. Ultrasound criteria for diagnosing PCOS include a transvaginal scan showing 12 or more follicles measuring 2–9 mm throughout the entire ovary or an ovarian volume ≥ 10 mL, though this is not recommended for women less than 8 years post-menarche [7]. The guidelines also emphasize using phenotype descriptions, identifying four phenotypes based on the presence or absence of the diagnostic criteria: Phenotype A (androgen excess, ovulatory dysfunction, and polycystic ovarian morphology), Phenotype B (androgen excess and ovulatory dysfunction, without polycystic ovarian morphology), Phenotype C (androgen excess and polycystic ovarian morphology, without ovulatory dysfunction), and Phenotype D (ovulatory dysfunction and polycystic ovarian morphology, without androgen excess) [8]. While PCOS is often associated with reproductive issues, it is crucial to understand that it also significantly increases the risk of metabolic disorders, including dyslipidemia which can lead to severe cardiovascular problems in the future [9,10]. To mitigate these risks, all women diagnosed with PCOS should have their lipid profile tested at diagnosis, with subsequent measurements based on signs of hyperlipidemia or other cardiovascular risk factors. Annual blood pressure monitoring is essential, particularly when planning pregnancy or fertility treatment, given the elevated risk of hypertensive disorders. Moreover, increased research funding is needed for PCOS, considering its high prevalence and wide-ranging impact on various health systems, including metabolic and cardiovascular health. A key feature of PCOS is insulin resistance, which significantly elevates the risk of developing impaired fasting glucose, impaired glucose tolerance, and type 2 diabetes, irrespective of a woman’s age or body mass index (BMI) [11]. Studies show that insulin resistance is present in 75% of women with normal BMI and 95% of those with a higher BMI [5]. Therefore, glycemic status should be assessed at diagnosis and reassessed every 1–3 years, depending on individual risk factors. Another condition frequently linked to PCOS is Obstructive Sleep Apnea (OSA). Women with PCOS should be assessed for symptoms such as snoring, waking up unrefreshed, daytime sleepiness, or fatigue [6]. Furthermore, women with PCOS face a higher risk of developing endometrial hyperplasia, which can progress to endometrial cancer. Although the overall risk of endometrial cancer remains low, routine screening for all women with PCOS is not advised [6]. Psychiatric disorders are common, often resulting from hormonal imbalances, body image concerns, and reproductive challenges. Depression, anxiety, eating disorders, and low self-esteem are prevalent and linked to the physical symptoms of PCOS, such as hirsutism, acne, and infertility, which can significantly impact a woman’s mental health. Studies indicate that women with PCOS are more likely to experience these psychological conditions compared to the general population [12,13]. Addressing mental health concerns should be an integral part of managing PCOS. Women with depression, anxiety, or eating disorders should be offered psychological therapy, such as cognitive behavioral therapy (CBT), in accordance with general population guidelines and patient preferences. In more severe cases, antidepressants or anxiolytics may be considered, especially when mental health issues are persistent [6]. Furthermore, it’s important to acknowledge that the psychological impact of PCOS extends beyond mental health issues. Many women diagnosed with PCOS experience significant lifestyle changes, particularly in diet, exercise, and health behaviors, as they attempt to manage their symptoms [14,15]. Research has shown that women with PCOS tend to have worse eating habits than women without the condition, with a higher intake of simple carbohydrates, saturated fats, and polyunsaturated fatty acids (PUFAs), and a lower intake of healthy fats and fiber. These dietary habits are associated with higher testosterone levels and greater disease severity [16]. The frustration of not seeing quick results from a healthy diet can also be demotivating, emphasizing the importance of encouraging patients to maintain a balanced lifestyle to improve metabolic and clinical outcomes. Interestingly, rapid weight loss can lead to improved depressive symptoms, showing how lifestyle changes can positively influence both physical and mental health [17]. Conversely, lifestyle disruptions and the struggle to manage weight and other symptoms can worsen depressive symptoms, creating a vicious cycle.

## 2. Objectives

This research paper aims to understand how changing eating habits at the time of PCOS suspicion can affect metabolic parameters of patients. Additionally, this study aims to identify potential interventions that may help women achieve better clinical and laboratory outcomes and it aimed to assess whether the mere suspicion of polycystic ovary syndrome could lead to modifications in lifestyle behaviors. Finally, it should be determined whether past or recent lifestyle changes made by women with PCOS translate into the results of laboratory, anthropological and clinical tests so the analyzes considered changes that had been maintained for a longer time (at least 6 months), recent changes and no changes in eating habits.

## 3. Material and Methods

### 3.1. Methods

A survey study was planned, in which women hospitalized in the Department of Gynecological Endocrinology of the Medical University of Silesia in Katowice who are suspected of having PCOS could take part. The term suspicion of the PCOS refers to patients referred from an outpatient clinic for further diagnosis of PCOS in a clinical setting. Patients in whom diagnosis PCOS was not ultimately confirmed were excluded from the study. The questionnaire was developed by the authors of this work between January 2023 and January 2024, and it comprised inquiries regarding fundamental details such as age and symptoms, along with inquiries about modifications in patients’ lifestyle, calorie consumption, and the glycemic index of their diet [Supplementary Data]. The Ethics Committee of the Medical University of Silesia approved the use of retrospective data from the patients’ files, no BNW/NWN/0052/KB/269/24. All patients gave written informed consent, and their confidentiality and anonymity were protected.

**Figure 1:**
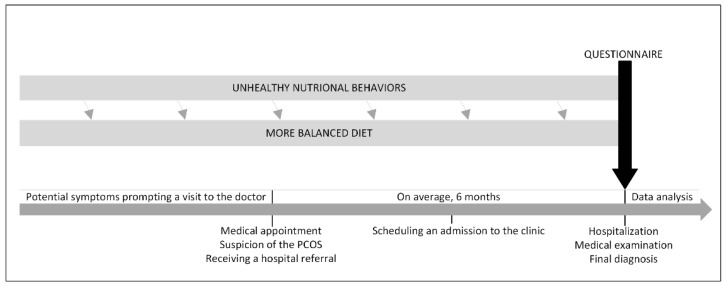
The moment of completing the questionnaire depending on the PCOS diagnosis process.

The Figure 1 illustrates timing of questionnaire completion by patients and the sequence of events leading to diagnostic confirmation. On average, the time from initial suspicion of the disease to its definitive diagnosis was six months. Therefore, lifestyle modifications implemented within this period may be associated with the suspicion of the condition itself.

The original questionnaire was developed by the study authors based on established guidelines regarding PCOS management [18,19]. The survey assessed the presence of various symptoms, including infrequent periods, absence of periods, frequent periods, acne, oily skin and hair, excessive male-pattern hair growth, hair loss in the central part of the scalp/temporal regions and difficulties in maintaining a healthy body weight, overweight, or obesity. The questionnaire was designed to include simple questions that would be easily understood by patients and related to basic dietary modifications. Patients were asked about reducing their consumption of sweets, red fatty meat, soft drinks, and alcohol and the response options included: “Yes, I started more than 6 months ago”, “Yes I started less than 6 months ago”, “No”, and “I have increased consumption”. The survey also examined the predominant dietary pattern of participants both more than 6 months ago and within the past 6 months. Respondents were asked to indicate whether their diet was primarily composed of high-glycemic or low-glycemic foods. Furthermore, the questionnaire collected data on current daily caloric intake as well as caloric intake 6 months prior. Participants could select one of the following options: 1200 kcal, 1500 kcal, 1800 kcal, 2000 kcal, 2500 kcal, 3000 kcal, “I did not count the daily caloric intake of my diet”, or they could specify their exact daily caloric intake (Supplementary Material—Questionnaire).

The source of information on the glycemic index of the foods listed in the questionnaire comes from the Systematic Review of International Tables of Glycemic Index and Glycemic Load Values 2021, which provides glycemic index values for over 4000 food products [20].

### 3.2. Patients

The study included patients aged 18–35 who were initially suspected and finally diagnosed with PCOS during hospitalization at the Department and Clinic of Gynecological Endocrinology of the Medical University of Silesia in Katowice. Diagnosis of PCOS was based on the presence of two out of three Rotterdam Criteria: ovulatory dysfunction presenting as irregular menstrual cycles or amenorrhea, clinical and/or biochemical hyperandrogenism, and ultrasound assessment of polycystic ovaries (≥12 antral follicles in one ovary or ovarian volume ≥ 10 mL) [7,21]. Clinical hyperandrogenism was defined as the presence of hirsutism (most sensitive indicator), acne, or alopecia. The Ferriman-Gallwey scoring system enables the definition and semi-quantitative assessment of hirsutism. This method evaluates nine body areas with androgen-sensitive hair (upper lip, chin, chest, upper back, lower back, upper abdomen, lower abdomen, upper arms, thighs), assigning a score from 1 (minimal terminal hair growth) to 4 (pronounced virilization). The scores for each assessed region are summed, with a total score of 8 or higher indicating the presence of hirsutism [22]. Biochemical hyperandrogenism refers to serum total testosterone > 0.481 ng/mL, free testosterone > 6.3 pg/mL, and/or free androgen index > 5%. The exclusion criterion for participation in the study was the occurrence of diseases that could cause similar symptoms, such as congenital adrenal hyperplasia, androgen-secreting tumors, Cushing’s syndrome, hypogonadotropic hypogonadism, premature ovarian failure, hyperprolactinaemia and thyroid dysfunction. Patients who were not diagnosed with PCOS during hospitalization were excluded from the study [7]. Gynecological ultrasound was performed with the use of Voluson E8 Expert (GE Healthcare, New York, NY, USA).

### 3.3. Measurements

Height and weight were measured in each patient. BMI was calculated as weight (kg) divided by the square of height (m^2^). The assessment of insulin resistance was carried out using the indirect method using the HOMA-IR index calculated from the formula:

HOMA-IR = fasting serum insulin concentration (μIU/mL) × fasting serum glucose concentration (mmol/L)/22.5.

Blood samples were taken after overnight fasting, between the second and fifth day of the menstrual cycle.

### 3.4. Biochemical Analyses

Serum concentrations of TSH, FT4, total testosterone, SHBG, FSH, LH, prolactin, dehydroepiandrosterone sulfate, and cortisol were determined by electrochemiluminescence “ECLIA” using Roche reagents on a Cobas 601 analyzer (Roche Diagnostics GmbH, Mannheim, Germany). Serum glucose, total cholesterol, HDL and LDL cholesterol, and triglycerides were deter-mined by colorimetry (AU 640 analyzer) using Beckman Coulter reagents (Brea, CA, USA). Insulin concentrations were determined using the chemiluminescence method (CMIA) using Abbott reagents (Alinity instrument; Chicago, IL, USA). The concentration of 17-hydroxyprogesterone and free testosterone was determined using the enzyme immunoassay ELISA from NovaTec (NovaTec Immunodiagnostica GmbH, Dietzenbach, Germany) on a Virclia analyzer (Diamedica, Riga, Latvia). The concentra- tion of androstenedione was determined with the immunochemical test using the chemiluminescence method “CLIA” using Siemens reagents (Immulite 2000 XPi apparatus; Siemens Healthcare GmbH, Erlangen, Germany).

### 3.5. Statistical Analysis

It was investigated whether particular types of self-declared changes in the lifestyle of women with PCOS affected different values of laboratory, clinical and anthropometric parameters. Among the parameters studied: scores in Ferriman-Gallwey scale and Global Acne Severity (GEA) scale, waist to hip ratio (WHR), BMI, systolic blood pressure, diastolic blood pressure, HOMA-IR and concentrations in the blood of: total cholesterol, high density lipoprotein (HDL), low density lipoprotein (LDL), triglycerides, thyroid stimulating hormone (TSH), free thyroxine (FT4), sex hormone binding globulin (SHBG), anti-Mullerian hormone (AMH), total testosterone (TT), free testosterone (TF), androstenedione (A4) only FT4, systolic blood pressure and diastolic blood pressure was normally distributed (Shapiro-Wilk test > 0.05) and the t-student test was used to examine their relationships. The remaining data were tested using Mann Whitney U test, because of a non-normal distribution of them. The significance level of 0.05 was adopted for the analysis and the STATISTICA 13.3 PL software (TIBCO Software Inc.) was used for all statistical analysis.

## 4. Results

Anthropometric and metabolic characteristics of the patients are presented in the table (Table 1). The tests were performed at the time of completing the questionnaire after dietary interventions. Among the 154 patients diagnosed with PCOS, classical phenotype A, with all three criteria fulfilled, was detected in 119 patients (77.3%), phenotype B in 13 patients (8.4%), phenotype C in 16 patients (10.4%), and phenotype in 6 patients (3.9%).

### 4.1. Limiting the Consumption of Specific Products

The women were asked about limiting the consumption of sweets, red and fatty meat, sweet sodas and nectars, and alcohol. Respondents could answer “yes, I started limiting more than 6 months ago”, “no”, “increased consumption” and” yes”, I started limiting less than 6 months ago”. The ultimate results are presented in Table 2.

It was determined to examine whether the self-reported reduction in consumption of unhealthy foods and alcohol affected the parameters listed in Table 2. Therefore, the patients were categorized into three groups based on each of the four restricted food and drink types (sweets, soft drinks, red, fatty meat and alcohol), and their laboratory and clinical parameters were analyzed. The classification, along with the mean and median values of the parameters, is detailed in the Supplementary Material—Document S2: Tables comparing the results of the parameters in patients depending on the reduction in the consumption of certain types of food and drink. Below, only the data demonstrating statistically significant differences between the groups are presented. If we compare women who made the changes more than 6 months ago versus those who did not reduce foods such as sweet and fatty red meat and alcohol, lower levels of total testosterone are noticeable (*p* < 0.05). The exact mean values, medians, and *p*-values are presented in the Table 3.

In women who reported reducing their consumption of red and fatty meat less than 6 months ago, higher average A4, AMH and acne (*p* < 0.05) were observed compared to those who did not implement such restrictions. Additionally, we observed higher FG score in women who have recently started to limit sweets and alcohol in their diet compared to women who admit that they do not limit these products. Comparing patients who limited their consumption of sugary drinks < 6 months with those who did not, higher average values (*p* < 0.05) of BMI, TG, FT4, SBP and TF were noted, as well as a lower average level of SHBG (*p* < 0.05). The precise mean values, medians, and *p*-values are shown in the Table 4.

Then we analyzed thoroughly patients who made dietary restrictions more than 6 months ago and those who had implemented such changes less than 6 months ago. In the group of women who reduced their red meat intake < 6 months ago, compared to those who had previously reduced their intake, higher mean values (*p* < 0.05) of A4, TT, and TF were observed. For women who reduced their consumption of sweets less than 6 months ago, higher mean values (*p* < 0.05) of BMI, FG score, TG and TF were recorded compared to the group that had made this change earlier. The group that reduced alcohol consumption < 6 months, compared to the group that had previously limited alcohol intake, showed higher mean values (*p* < 0.05) of FG score, TT and TF. For women who reduced soft drinks consumption < 6 months ago, higher mean values (*p* < 0.05) of FG score, BMI, TG, insulin, TT, TF, SBP were noted, as well as lower mean values (*p* < 0.05) of SHBG and HDL-C, compared to patients who had previously reduced soft drinks consumption. The precise mean values, medians, and *p*-values are shown in the Table 5.

### 4.2. The Glycemic Index of the Diet Followed Before 6 Months Ago and After

Respondents were asked whether foods with a high or low glycemic index predominate in their diet. 54 (35.1%) of them reported eating mainly high glycemic index foods (such as processed grain products, white bread, sweets, potatoes, bananas, sweet corn) both before 6 months ago and in recent 6 months. Meanwhile, 43 (27.9%) women indicated that they consistently consumed mostly low glycemic index foods (such as fresh fruits, green vegetables, legumes, low-fat dairy products, lean meats, fish, rye bread, and groats.) for over 6 months. A significant portion, 56 (37%) women, reported changing their diet from high to low glycemic index foods within 6 months. Interestingly, despite the different dietary approaches among these groups of women, there were no statistically significant changes in the biochemical and metabolic parameters listed in Table 2.

### 4.3. Daily Caloric Intake Before and After the Suspicion of PCOS

The respondents were asked about their daily caloric intake before 6 months and now. The range of responses regarding caloric intake included from 1200 to 3000 kcal. Additionally, patients could respond that they did not count or do not count calories, or they could provide a different caloric value for their diet.

There were 65 patients (42.2%) who counted calories both before 6 months ago and now, while another 65 (42.2%) at all. Among the respondents, there was also a group of 24 women (15.6%) who started counting calories in the last 6 months.

A change was observed in the 120-min glucose tolerance test between women who had counted calories for more than 6 months and those who reported never counting calories. Specifically, among women who had counted calories, the 120-min glucose tolerance test level averaged 108.83 mg/dL (SD: 29.2, median: 104.5), whereas among women who did not count calories, the average was 122.99 mg/dL (SD: 33.32, median: 111) with *p* = 0.02.

## 5. Discussion

A large percentage of women have made changes in their lifestyle. Less than half of women did not limit harmful foods, products with a high glycemic index, and ignored the number of calories. The distinction between the group of women who introduce particular dietary changes recently (up to 6 months) and those who maintain these changes for a longer time seems to be justified because of large differences in clinical and laboratory parameters. In women with PCOS, studies suggest that an appropriate diet, such as the DASH diet and other calorie-restricted diets, has a beneficial effect on handling insulin resistance (IR) and body weight management [23]. The results show that women who reduced the intake of red meat and sweets over 6 months ago had significantly lower levels of total testosterone (TT) and other androgens. This is particularly relevant as hyperandrogenism is a hallmark feature of PCOS. This suggests that dietary changes, particularly reducing foods high in saturated fats and simple sugars, can positively influence the hormonal balance in PCOS. Interestingly, a direct correlation has been noted between adherence to the Mediterranean diet and indicators of disease severity. In women with PCOS, an increased intake of simple carbohydrates and saturated fatty acids (SFA), along with a low intake of complex carbohydrates, fiber, and monounsaturated fatty acids (MUFA), was observed compared to the control group. This dietary pattern was associated with worsening parameters such as hyperandrogenemia, inflammation, and insulin resistance [16]. Addressing obesity through lifestyle changes is a crucial strategy for treating PCOS, as it enhances insulin sensitivity and improves both reproductive and metabolic features [24]. One of the key findings of this study is that alcohol reduction is associated with improvements in metabolic and hormonal parameters in women with PCOS. Specifically, participants who reduced alcohol intake for over six months showed lower levels of total testosterone and Ferriman-Gallwey scores, which suggests decreased androgenic activity. The data underscores the importance of long-term lifestyle adjustments for improving PCOS-related health outcomes, as recent or inconsistent alcohol reduction appears insufficient for achieving optimal metabolic and hormonal balance. However, awareness around these effects remains limited, and many women with PCOS are not fully informed about how even moderate alcohol consumption can hinder their health outcomes. Raising awareness is essential to empower women with PCOS to make informed choices that support their well-being.

Interestingly, the study found that 37% of women had transitioned to a lower glycemic index (GI) diet within the past six months. This finding suggests a thorough understanding of PCOS and an awareness that diet is a crucial factor in alleviating the course of the disease. However, despite this, no significant changes were observed in the clinical or biochemical parameters listed in Table 2. In contrast a systematic search through major indexing databases, including Scopus, Pubmed/Medline, ISI web of science, Embase, Cochrane central, and CINAHL (1966–30 April 2021) shows that lower glycemic index play a significant role in reducing the risk and improving the clinical and biochemical features of PCOS [25]. The lack of significant findings in this study may be because of the short timeframe in which these dietary changes were implemented or variations in the adherence to the low glycemic index diet. The problem also might stem from a lack of knowledge about this type of diet. When specific product restrictions were asked, there were statistically significant results, suggesting that understanding was clearer. It may also be essential to provide patients with clear, well-defined goals. A straightforward guideline, such as excluding sweets from the diet, might be more effective for patients than advising them to follow a low glycemic index diet, which could be less immediately understandable. This conclusion stands in line with the SMART method used for effective goal setting [26]. According to the SMART method the aim should be specific, measurable, attainable, relevant, and time-bound (SMART). It has been demonstrated that nutritional mistakes made by women with PCOS contribute to metabolic disorders that impair proper ovarian function, emphasizing the importance of health education in the field of nutrition [27]. Moreover, the study showed that women who recently reduced their intake of unhealthy foods had worse outcomes, such as higher BMI, triglycerides, and androgen levels, compared to those who made these changes earlier, which may indicate the need for a long-term diet to gain metabolic benefits. It’s also essential to address the BMI differences among the patient groups. Those who made dietary changes for less than six months may have a higher BMI than those who did not alter their diet. This raises the issue of not having baseline data before dietary changes, limiting our conclusions and indicating a potential weakness in the study. Furthermore, since this is a heterogeneous group, patients with lower BMI may lack motivation for healthy lifestyle changes. The findings clearly demonstrate that BMI significantly affects metabolic and hormonal parameters in PCOS, with lower-BMI patients showing better outcomes without dietary modifications.

An interesting finding is that women who counted calories for over six months did not achieve statistically significant changes in biochemical parameters. This suggests that calories counting alone does not lead patients to change their eating habits or lose weight. Without clear dietary recommendations, such as reducing the intake of sweets or alcohol, calories counting alone is ineffective.

Notably, patients who have not reduced junk food consumption exhibit less severe acne and lower testosterone levels than those who recently changed their diet. This paradox suggests recent dietary changes may be associated with greater clinical severity of acne. It is important to note that a characteristic feature of PCOS is hyperandrogenemia, which is a key factor contributing to occurring acne. Its levels can have a dominant influence on the severity of skin lesions [28]. One explanation is that sudden dietary changes, particularly reductions in high-glycemic foods, may initially cause temporary hormonal fluctuations as the body adapts. This adjustment period could manifest as transient increases in testosterone levels, leading to a short-term rise in symptoms like acne. Individuals who have not reduced junk food intake may also have other lifestyle factors, such as exercise habits or stress levels, that influence their acne. These factors might mitigate the effects of a high-junk-food diet, resulting in less severe symptoms.

Hirsutism is typically a sign of elevated androgen levels. Hair length depends on the duration of the anagen phase and the rate of growth, which vary depending on the hair type and body area. Since the anagen cycle lasts approximately four months, a period of about six months is required to observe changes [29]. This translates into potential unreliability in assessing the severity of hirsutism among the patients we studied. A comparison between women with PCOS and a healthy control group revealed that women with PCOS have a lower overall diet quality, poorer quality of consumed foods (e.g., higher cholesterol levels), and a lower total level of physical activity compared to women without PCOS [30]. Another perspective considers the role of psychological and physiological stress. For patients attempting to reduce junk food, especially if done abruptly or as part of a restrictive diet, stress levels might rise. Elevated stress is known to influence androgen production, potentially aggravating conditions like acne and hirsutism. Moreover, stress-induced shifts in cortisol and insulin levels could further disrupt androgen balance, which might be especially impactful in individuals predisposed to hormonal fluctuations. Lastly, those who haven’t changed their diet might have unknowingly adapted to a hormonal baseline shaped by their long-standing eating habits. This “stability” could make them less susceptible to fluctuations, even if their diet includes more high-glycemic or processed foods. This observation highlights the complex and often non-linear relationship between diet, hormonal regulation, and skin health, underscoring the need for a holistic approach when assessing dietary impacts on dermatological and hormonal conditions.

An important fact is that women with PCOS encounter heightened levels of anxiety, depression, and body image concerns, which can drive substantial changes in their lifestyle choices [14,15]. These changes often include alterations in diet, exercise habits, and general health behaviors as they attempt to manage or mitigate their symptoms. On the other hand, depression can lead to poor adherence to healthy lifestyle behaviors, such as maintaining a balanced diet and regular physical activity, which are crucial for managing PCOS symptoms [31,32] Given the association between PCOS and a heightened risk of moderate to severe depression and anxiety symptoms, it appears that doctors should contemplate screening women with PCOS for these conditions [33]. Therefore, it seems that a large percentage of women with severe clinical symptoms express a desire to change their lifestyle and it is necessary to constantly motivate and evaluate the motivation and self-determination of the patient to maintain a healthy diet in order to get the expected changes such as improvements in clinical and laboratory parameters and better mental state. Also, it is very important to mention that early diagnosis, health education and motivating patients to maintain a healthy lifestyle are important to prevent metabolic complications [24,34]. A study conducted in Canada suggests that to improve care for women with PCOS, knowledge and awareness of the disease should be increased among both primary care physicians and the public. Additionally, the importance of conducting research on PCOS, increasing the number of specialists, and providing reliable sources of information and support for patients was emphasized [35].

A strength of the study was the direct access to patients; the survey was conducted in a clinical setting, which facilitated easy contact with respondents. The data collection occurred from January 2023 to January 2024, ensuring the relevance of the results. Patients completed the survey in the hospital after all their concerns were addressed by qualified personnel, which enhanced the credibility and accuracy of the study. Thanks to direct contact with patients, we could make sure that they understood all the aspects raised in the survey and help them answer questions more precisely if they had any doubts. Surveys were completed for all patients at the diagnostic stage, allowing access to a large number of respondents at various stages of the disease. Each patient was diagnosed at the same clinical center of reference, following the same criteria. The study enables the analysis of changes in health behaviors over time, providing information about the subjective experiences and feelings of patients both before and after the diagnosis of PCOS. Another strength of the study was its reliance on parameters directly related to PCOS, resulting in high specificity. Additionally, our research presents a multidisciplinary approach to the patients studied, considering various psychological factors. A novel aspect of this study is its simplicity, focusing on how implementing basic changes influences biochemical and metabolic parameters over both short and long periods, making the study’s findings potentially motivational for other patients with PCOS. Our study helps us to understand the overall trends in the PCOS patient population, which can be used to develop better patient support programs.

The weaknesses of the study arise from the subjectivity of the responses, which may be distorted by personal feelings, current well-being, and the health status of the patients. Additionally, the surveys were completed in a hospital setting, where factors such as medical procedures, interaction with healthcare staff, and being away from home could create stressful conditions for the patients, potentially affecting the results. Some complex factors influencing lifestyle make it difficult to draw definitive conclusions; furthermore, patients’ lifestyles may have changed over time depending on various circumstances, which could lead to challenges in providing accurate responses. The time elapsed was too short to fully observe changes in some of the evaluated parameters. Unfortunately, we do not have access to the patients’ results corresponding to the period prior to implementing lifestyle changes. Moreover, following health-promoting changes is influenced by broader social and cultural contexts, which are difficult to account for in research.

## 6. Conclusions

The findings of this study highlight the importance of sustained dietary modifications in managing PCOS. However, it should be emphasized that lifestyle interventions do not alter the diagnosis itself. Nevertheless, they can aid in symptom management and contribute to the restoration of certain metabolic and hormonal parameters, primarily through improvements in body mass index. Weight loss through diet, physical activity, and lifestyle changes in women with PCOS, especially those with obesity, can improve metabolic parameters, reduce the risk of type 2 diabetes, lower triglycerides, and enhance insulin sensitivity. It also helps normalize hormone levels, alleviating symptoms like hirsutism and acne, supports ovarian function and menstrual regularity [36]. Long-term changes, particularly reducing red meat, sweets, and alcohol, were associated with better hormonal and metabolic profiles, while short-term changes showed limited benefit. It should also be emphasized that the mere suspicion of PCOS can act as a motivator for lifestyle changes, and in some women, it prompts alterations in their diet. Suspected PCOS led to dietary modifications in more than half of the surveyed patients. The reduction in the consumption of foods such as sweets, high-fat red meat, soft drinks, and alcohol, depending on the timing of dietary changes (more than six months ago or within the past six months), was associated with significant changes in parameters including BMI, systolic blood pressure, serum lipid levels, fasting glucose and insulin concentrations, sex hormone-binding globulin, total testosterone, free testosterone, androstenedione, and acne severity. It is particularly important to note that women who had reduced intake of these products for more than 6 months exhibited significantly lower total testosterone levels compared to those who did not restrict these products (*p* < 0.05). Specifically, mean TT levels were 0.375 ± 0.18 ng/mL vs. 0.787 ± 2.19 ng/mL (*p* = 0.008) for red meat, 0.359 ± 0.18 ng/mL vs. 0.681 ± 1.9 ng/mL (*p* = 0.02) for sweets, and 0.388 ± 0.19 ng/mL vs. 0.917 ± 2.65 ng/mL (*p* = 0.004) for alcohol. The study revealed that women who had only recently reduced their consumption of unhealthy foods exhibited less favorable outcomes, including higher BMI, triglyceride levels, and androgen concentrations, compared to those who implemented these dietary changes earlier. This suggests that sustained dietary modifications may be necessary to achieve meaningful metabolic benefits. These results underscore the need for healthcare providers to emphasize the importance of long-term dietary adherence and provide ongoing support to women with PCOS as they navigate these lifestyle changes. No statistically significant correlations were observed in women transitioning from a high-glycemic index diet to a low-glycemic index diet. This may be due to the short duration of dietary changes or variations in adherence to a low-glycemic index diet. Another possible explanation is a lack of knowledge about this type of diet. Additionally, no statistically significant correlations were observed in women practicing calorie counting. These findings suggest that calorie counting alone is insufficient to modify patients’ dietary habits or promote weight loss. Without clear dietary guidelines, such as reducing the intake of sweets or alcohol, calorie counting appears to be ineffective. This study highlights the importance of establishing clearly defined dietary goals, such as restricting specific food types, rather than setting broad and complex nutritional objectives. Empowering patients through education, early diagnosis, and continuous motivation can lead to better outcomes, not just in managing PCOS symptoms but also in preventing related metabolic complications.

## Figures and Tables

**Table 1:** Anthropometric metabolic and hormonal characteristics of the patient with PCOS (*n* = 154).

Parameter	Mean ± SD	Median
Age (years)	25.1 ± 5.1	24
BMI (kg/m^2^)	26.1 ± 6.16	24.45
WHR	0.8 ± 0.08	0.79
TC (mg/dL) (<190)	173.56 ± 33.89	171
HDL-C (mg/dL) (>40)	55.79 ± 13.45	55.75
LDL-C (mg/dL) (<135)	98.57 ± 27.51	94.4
TG (mg/dL) (<150)	101.32 ± 54.56	87.9
TSH (µIU/mL) (0.27–4.2)	1.87 ± 1.05	1.65
FT4 (ng/dL) (0.93–1.71)	1.22 ± 0.15	1.22
Fasting Glucose (mg/dL) (70–99)	84.80 ± 6.44	84
Glucose 120 min OGTT (mg/dL) (<140)	114.38 ± 31.24	108
Fasting INS (µU/mL) (2.6–24.9)	9.28 ± 6.07	7.35
HOMA-IR	1.98 ± 1.39	1.54
SBP (mmHg) (<140)	124.81 ± 11.5	125
DBP (mmHg) (<90)	78.58 ± 9.3	79
SHBG (nmol/L) (32.4–128)	50.11 ± 29.63	43.85
AMH (ng/mL) (1.2–9.05)	6.56 ± 4.02	5.41
TT (ng/mL) (0.084–0.481)	0.53 ± 1.33	0.37
TF (pg/mL) (0.1–6.3)	2.74 ± 2.6	2.03
A4 (ng/mL) (0.49–1.31)	1.65 ± 0.66	1.5

(BMI—body mass index; WHR—waist to hip ratio; TC—Total cholesterol, HDL-C—high-density lipoprotein cholesterol; LDL-C—low-density lipoprotein cholesterol; TG—triglycerides; TSH—thyroid-stimulating hormone; FT4—free thyroxine; INS—insulin; HOMA-IR—homeostatic assessment of insulin resistance; SBP—Systolic blood pressure; DBP—Diastolic blood pressure; SHBG—sex hormone binding globulin; AMH—anti-Mullerian hormone; TT—total testosterone; TF—free testosterone, A4—androstendione).

**Table 2:** Number and percentage of women declaring reduction and lack of reduction in the intake of some types of food and drink.

Type of Reduced Food/Drink	Reduction Intake				No Reduction	
	<6 Months Ago		>6 Months Ago			
	*n*	Percentage of All	*n*	Percentage of All	*n*	Percentage of All
sweets	54	35%	28	18.2%	72	46.8%
red, fatty meat	28	18.2%	75	48.7%	51	33.1%
soft drinks	49	31.8%	71	46.1%	34	22.1%
alcohol	25	16.2%	91	59.1%	38	24.7%

**Table 3:** Statistically significant results comparing women with PCOS who reduced the amount of specific types of food more than 6 months ago compared to those who did not restrict them.

Type of Reduced Food/Drink	Parameters	Reduction of the Intake >6 Months Ago		No Reduction of the Intake		*p* Value
		Mean ± SD	Median	Mean ± SD	Median	
red meat	TT	0.375 ± 0.18	0.34	0.787 ± 2.19	0.39	0.008
sweets	TT	0.359 ± 0.18	0.335	0.681 ± 1.9	0.4	0.02
alcohol	TT	0.388 ± 0.19	0.34	0.917 ± 2.65	0.425	0.004

(TT—total testosterone).

**Table 4:** Statistically significant results comparing women with PCOS who reduced the amount of specific types of food less than 6 months ago compared to those who did not restrict them.

Type of Reduced Food/Drink	Parameters	Reduction of the Intake <6 Months Ago		No Reduction of the Intake		*p* Value
		Mean ± SD	Median	Mean ± SD	Median	
red meat	acne	1.43 ± 0.96	1	1 ± 1.13	1	0.047
	AMH	8 ± 4.9	6.5	6 ± 0.38	4.9	0.04
	A4	2.04 ± 0.83	1.88	1.6 ± 0.6	1.5	0008
sweets	FG	7.48 ± 5.54	6.5	6.9 ± 10	3	0.04
alcohol	FG	8.6 ± 6.8	6	7.58 ± 13	3	0.04
soft drinks	BMI	28.1 ± 6.2	26.35	25.65 ± 6.61	24	0.04
	SHBG	40.24 ± 23.52	36.3	49.9 ± 22.3	5.65	0.03
	FT4	1.24 ± 0.14	1.26	1.17 ± 0.13	1.17	0.04
	TF	3.6 ± 3.4	3	2.35 ± 2.28	1.65	0.03
	SBP	128.5 ± 9.8	128	122.6 ± 13.3	123	0.04

(BMI—body mass; FT4—free thyroxine; SBP—Systolic blood pressure; SHBG—sex hormone binding globulin; AMH—anti-Mullerian hormone; TF—free testosterone, A4—androstenedione; FG—assessment of hirsutism on the Ferriman-Gallwey scoring system).

**Table 5:** Statistically significant results comparing women with PCOS who reduced the amount of specific types of food over 6 months ago compared to those who did it less than 6 months ago.

Type of Reduced Food/Drink	Parameters	Reduction of the Intake >6 Months Ago		Reduction of the Intake <6 Months Ago		*p* Value
		Mean ± SD	Median	Mean ± SD	Median	
red meat	TT	0.375 ± 0.18	0.34	0.499 ± 0.22	0.51	0.009
	TF	2.31 ± 2.67	1.55	3.266 ± 2.07	2.94	0.0048
	A4	1.54 ± 0.57	1.43	2.04 ± 0.83	1.88	0.001
sweets	FG	5.18 ± 5.9	3	7.48 ± 5.54	6.5	0.039
	BMI	23.58 ± 5.2	23.3	27.3 ± 6.1	26.46	0.012
	TG	89.36 ± 57	77.05	107.3 ± 50.4	98.6	0.028
	TF	1.89 ± 1.49	1.48	2.97 ± 2.18	2.235	0.034
alcohol	FG	5.97 ± 5.9	4	8.6 ± 6.8	6	0.04
	TT	0.388 ± 0.19	0.34	0.486 ± 0.21	0.47	0.027
	TF	2.384 ± 2.32	1.63	3.54 ± 3.43	2.77	0.013
soft drinks	FG	5.72 ± 6.86	3	7.96 ± 6.28	8	0.012
	BMI	24.91 ± 5.64	23.78	28.1 ± 6.17	26.35	0.0048
	SHBG	57.02 ± 34.46	53.7	40.24 ± 23.52	36.3	0.008
	HDL-C	58.85 ± 14.86	58.8	51.33 ± 12.1	50.2	0.0049
	TG	95.1 ± 54.9	77.3	112.2 ± 50.3	103	0.007
	INS	8.43 ± 5.41	7.1	10.72 ± 6.72	8.45	0.04
	TT	0.386 ± 0.162	0.36	0.499 ± 0.239	0.47	0.019
	TF	2.36 ± 2.01	1.93	3.61 ± 3.36	3.04	0.023
	SBP	123.3 ± 11.2	120	128.5 ± 9.8	128	0.006

(BMI—body mass index; FG—assessment of hirsutism on the Ferriman-Gallwey scoring system; HDL-C—high-density lipoprotein cholesterol; TG—triglycerides; INS—insulin; SBP—Systolic blood pressure; SHBG—sex hormone binding globulin; TT—total testosterone; TF—free testosterone, A4—androstenedione).

## Data Availability

Data are available upon reasonable request from the corresponding author.

## Supplementary Materials

The following supporting information can be downloaded at: https://www.mdpi.com/article/10.3390/jcm14082708/s1, Document S1: Questionnaire, Document S2: Tables comparing the results of the parameters in patients depending on the reduction in the consumption of certain types of food and drink.

## References

[1] Azziz R., Carmina E., Dewailly D., Diamanti-Kandarakis E., Escobar-Morreale H.F., Futterweit W., Janssen O.E., Legro R.S., Norman R.J., Taylor A.E. (2006). Position statement: Criteria for defining polycystic ovary syndrome as a predominantly hyperandrogenic syndrome: An Androgen Excess Society guideline. J. Clin. Endocrinol. Metab..

[2] Diamanti-Kandarakis E., Kandarakis H., Legro R. (2006). The role of genes and environment in the etiology of PCOS. Endocrine.

[3] March W., Moore V., Willson K., Phillips D., Norman R., Davies M. (2010). The prevalence of polycystic ovary syndrome in a community sample assessed under contrasting diagnostic criteria. Hum. Reprod..

[4] Bozdag G., Mumusoglu S., Zengin D., Karabulut E., Yildiz B.O. (2016). The prevalence and phenotypic features of polycystic ovary syndrome: A systematic review and meta-analysis. Hum. Reprod..

[5] Jeanes Y.M., Barr S., Smith K., Hart K.H. (2009). Dietary management of women with polycystic ovary syndrome in the United Kingdom: The role of dietitians. J. Hum. Nutr. Diet..

[6] Teede H.J., Tay C.T., Laven J., Dokras A., Moran L., Piltonen T., Costello M., Boivin J., Redman L., Boyle J. (2023). International Evidence-Based Guideline for the Assessment and Management of Polycystic Ovary Syndrome 2023.

[7] The Rotterdam ESHRE/ASRM-Sponsored PCOS Consensus Workshop Group (2004). Revised 2003 Consensus on Diagnostic Criteria and Long-Term Health Risks Related to Polycystic Ovary Syndrome (PCOS). Hum. Reprod..

[8] Sachdeva G., Gainder S., Suri V., Sachdeva N., Chopra S. (2019). Comparison of the Different PCOS Phenotypes Based on Clinical Metabolic, and Hormonal Profile, and their Response to Clomiphene. Indian J. Endocrinol. Metab..

[9] Zhang J., Xu J.H., Qu Q.Q., Zhong G.Q. (2020). Risk of Cardiovascular and Cerebrovascular Events in Polycystic Ovarian Syndrome Women: A Meta-Analysis of Cohort Studies. Front. Cardiovasc. Med..

[10] Lim S.S., Kakoly N.S., Tan J.W.J., Fitzgerald G., Bahri Khomami M., Joham A.E., Cooray S.D., Misso M.L., Norman R.J., Harrison C.L. (2019). Metabolic syndrome in polycystic ovary syndrome: A systematic review, meta-analysis and meta-regression. Obes. Rev. Off. J. Int. Assoc. Study Obes..

[11] Cassar S., Misso M.L., Hopkins W.G., Shaw C.S., Teede H.J., Stepto N.K. (2016). Insulin resistance in polycystic ovary syndrome: A systematic review and meta-analysis of euglycaemic–hyperinsulinaemic clamp studies. Hum. Reprod..

[12] Pastore L.M., Patrie J.T., Morris W.L., Dalal P., Bray M.J. (2011). Depression symptoms and body dissatisfaction association among polycystic ovary syndrome women. J. Psychosom. Res..

[13] Teede H.J., Misso M.L., Costello M.F., Dokras A., Laven J., Moran L., Piltonen T., Norman R.J. (2018). Recommendations from the international evidence-based guideline for the assessment and management of polycystic ovary syndrome. Hum. Reprod..

[14] Dokras A., Clifton S., Futterweit W., Wild R. (2012). Increased Prevalence of Anxiety Symptoms in Women with Polycystic Ovary Syndrome: Systematic Review and Meta-Analysis. Fertil. Steril..

[15] Pokora K., Kowalczyk K., Wikarek A., Rodak M., Pędrys K., Wójtowicz M., Wyskida K., Jonderko M. (2022). Depressive Symptoms and Control of Emotions among Polish Women with Polycystic Ovary Syndrome. Int. J. Environ. Res. Public Health.

[16] Barrea L., Arnone A., Annunziata G., Muscogiuri G., Laudisio D., Salzano C., Pugliese G., Colao A., Savastano S. (2019). Adherence to the Mediterranean Diet, Dietary Patterns and Body Composition in Women with Polycystic Ovary Syndrome (PCOS). Nutrients.

[17] Fabricatore A.N., Wadden T.A., Moore R.H., Butryn M.L., Heymsfield S.B., Nguyen A.M. (2009). Predictors of Attrition and Weight Loss Success: Results from a Randomized Controlled Trial. Behav. Res. Ther..

[18] Moran L.J., Pasquali R., Teede H.J., Hoeger K.M., Norman R.J. (2009). Treatment of Obesity in Polycystic Ovary Syndrome: A Position Statement of the Androgen Excess and Polycystic Ovary Syndrome Society. Fertil. Steril..

[19] Norman R.J., Teede H.J. (2018). A New Evidence-Based Guideline for Assessment and Management of Polycystic Ovary Syndrome. Med. J. Aust..

[20] Atkinson F.S., Brand-Miller J.C., Foster-Powell K., Buyken A.E., Goletzke J. (2021). International Tables of Glycemic Index and Glycemic Load Values 2021: A Systematic Review. Am. J. Clin. Nutr..

[21] Balen A.H., Laven J.S.E., Tan S.-L., Dewailly D. (2003). Ultrasound Assessment of the Polycystic Ovary: International Consensus Definitions. Hum. Reprod. Update.

[22] Hatch R., Rosenfield R.L., Kim M.H., Tredway D. (1981). Hirsutism: Implications, etiology, and management. Am. J. Obstet. Gynecol..

[23] Shang Y., Zhou H., Hu M., Feng H. (2020). Effect of Diet on Insulin Resistance in Polycystic Ovary Syndrome. J. Clin. Endocrinol. Metab..

[24] Teede H., Deeks A., Moran L. (2010). Polycystic Ovary Syndrome: A Complex Condition with Psychological, Reproductive and Metabolic Manifestations That Impacts on Health across the Lifespan. BMC Med..

[25] Saadati N., Haidari F., Barati M., Nikbakht R., Mirmomeni G., Rahim F. (2021). The Effect of Low Glycemic Index Diet on the Reproductive and Clinical Profile in Women with Polycystic Ovarian Syndrome: A Systematic Review and Meta-Analysis. Heliyon.

[26] Bjerke M.B., Renger R. (2017). Being smart about writing SMART objectives. Eval. Program Plan..

[27] Szczuko M., Sankowska P., Zapałowska-Chwyć M., Wysokiński P. (2017). Studies on the Quality Nutrition in Women with Polycystic Ovary Syndrome (PCOS). Rocz. Państwowego Zakładu Hig..

[28] Dadachanji R., Shaikh N., Mukherjee S. (2018). Genetic Variants Associated with Hyperandrogenemia in PCOS Pathophysiology. Genet. Res. Int..

[29] Matheson E., Bain J. (2019). Hirsutism in Women. Am. Fam. Physician.

[30] Kazemi M., Kim J.Y., Wan C., Xiong J.D., Michalak J., Xavier I.B., Ganga K., Tay C.T., Grieger J.A., Parry S.A. (2022). Comparison of Dietary and Physical Activity Behaviors in Women with and without Polycystic Ovary Syndrome: A Systematic Review and Meta-Analysis of 39,471 Women. Hum. Reprod. Update.

[31] Greenwood E.A., Pasch L.A., Cedars M.I., Legro R.S., Eisenberg E., Huddleston H.G. (2018). Insulin Resistance Is Associated with Depression Risk in Polycystic Ovary Syndrome. Fertil. Steril..

[32] Tan S., Hahn S., Benson S., Janssen O.E., Dietz T., Kimmig R., Hesse-Hussain J., Mann K., Schedlowski M., Arck P.C. (2008). Psychological Implications of Infertility in Women with Polycystic Ovary Syndrome. Hum. Reprod..

[33] Cooney L.G., Lee I., Sammel M.D., Dokras A. (2017). High Prevalence of Moderate and Severe Depressive and Anxiety Symptoms in Polycystic Ovary Syndrome: A Systematic Review and Meta-Analysis. Hum. Reprod..

[34] Goh J.E., Farrukh M.J., Keshavarzi F., Yap C.S., Saleem Z., Salman M., Ramatillah D.L., Goh K.W., Ming L.C. (2022). Assessment of Prevalence, Knowledge of Polycystic Ovary Syndrome and Health-Related Practices among Women in Klang Valley: A Cross-Sectional Survey. Front. Endocrinol..

[35] Ismayilova M., Yaya S. (2022). What Can Be Done to Improve Polycystic Ovary Syndrome (PCOS) Healthcare? Insights from Semi-Structured Interviews with Women in Canada. BMC Women’s Health.

[36] Ozgen Saydam B., Yildiz B.O. (2021). Weight management strategies for patients with PCOS: Current perspectives. Expert Rev. Endocrinol. Metab..

