# Supplementary material for: Dietary Patterns and Metabolic and Hormonal Parameters in Women with Suspected PCOS
Source: J Clin Med. 2025 Apr 15;14(8):2708. doi: 10.3390/jcm14082708 (PMC12027942; doi:10.3390/jcm14082708)
Supplement: Supplementary file 1 [file jcm-14-02708-s001.zip › Questionnaire.pdf]

Supplementary data - Document S1: Questionnaire

Questionnaire on Lifestyle, Nutrition, Symptoms in Women Suspected/Diagnosed with Polycystic Ovary Syndrome (PCOS)

I give my informed consent to participate in the study.
a) yes
b) no
How old are you? …………..
Since when were you suspected of having Polycystic Ovary Syndrome (PCOS)? ……..
If you have been diagnosed with PCOS, when did the diagnosis occur (PCOS)? ……..

I Please select all the symptoms you are experiencing.

Do you have…?	Yes	No
Infrequent periods (less frequent than every 35 days) / absence of periods		
Frequent periods (more often than every 21 days)		
Acne		
Oily skin and hair		
Excessive male-pattern hair growth (face, chest, abdomen, back, thighs)		
Hair loss in the central part of the scalp/temporal regions (receding hairline)		
Difficulty maintaining a healthy weight / Overweight or obesity		

Please check all the symptoms that prompted you to visit the doctor.

	Please select all the applicable answers below.
Infrequent periods (less frequent than every 35 days) / absence of periods	
Frequent periods (more often than every 21 days)	
Acne	
Oily skin and hair	
Excessive male-pattern hair growth (face, chest, abdomen, back, thighs)	
Hair loss in the central part of the scalp/temporal regions (receding hairline)	
Difficulty maintaining a healthy weight / Overweight or obesity	
Difficulty conceiving	
Cases of PCOS among family and friends	
Information about PCOS on social media	
PCOS was diagnosed incidentally during routine examinations or tests related to another condition	
Others	What?

II Do you reduce your consumption

1. Of sweets?
a) Yes, I started more than 6 months ago
b) Yes, I started less than 6 months ago
c) No
d) I have increased consumption

2. Of red and fatty meat?
a) Yes, I started more than 6 months ago
b) Yes, I started less than 6 months ago
c) No
d) I have increased consumption

3. Of sweet sodas and nectars?
a) Yes, I started more than 6 months ago
b) Yes, I started less than 6 months ago
c) No
d) I have increased consumption

4. Of alcohol?
a) Yes, I started more than 6 months ago
b) Yes, I started less than 6 months ago
c) No
d) I have increased consumption

III

1. What type of food dominated your diet more than 6 months ago?

a) Mainly high glycemic index foods (such as processed grain products, white bread, sweets, potatoes, bananas, sweet corn)

b) Mainly low glycemic index foods (such as fresh fruits, green vegetables, legumes, low-fat dairy products, lean meats, fish, rye bread, and groats.)

2. What type of food has dominated your diet in recent 6 months?

a)Mainly high glycemic index foods (such as processed grain products, white bread, sweets, potatoes, bananas, sweet corn)

b) Mainly low glycemic index foods (such as fresh fruits, green vegetables, legumes, low-fat dairy products, lean meats, fish, rye bread, and groats.)

IV
1. **What was the daily caloric intake of your diet more than 6 months ago?**

a) 1200 kcal
b) 1500 kcal

c) 1800 kcal
d) 2000 kcal
e) 2500 kcal
f) 3000 kcal
g) I did not count the daily caloric intake of my diet
h) Own answer …….

2. **What is the daily caloric intake of your diet now?**

a) 1200 kcal
b) 1500 kcal
c) 1800 kcal
d) 2000 kcal
e) 2500 kcal
f) 3000 kcal
g) I do not count the daily caloric intake of my diet
h) Own answer …….
