# Supplementary material for: Dietary Patterns and Metabolic and Hormonal Parameters in Women with Suspected PCOS
Source: J Clin Med. 2025 Apr 15;14(8):2708. doi: 10.3390/jcm14082708 (PMC12027942; doi:10.3390/jcm14082708)
Supplement: Supplementary file 1 [file jcm-14-02708-s001.zip › Supplementary Materials S2.pdf]

Tables comparing the results of the parameters in patients depending on the reduction in the consumption of certain types of food and drink.

Tables 1-4 below present the laboratory and clinical results of the patients, divided into patients who do not restrict, restrict for the less than 6 months and restrict more than 6 months sweets (Table 1), soft drinks (Table 2), read, fatty meat (Table 3) and alcohol (Table 4). Tables containing only data that are statistically significant differ between groups are presented in the main text.

Table 1. Parameters of patients divided into three groups: not reducing sweet in their diet, reducing for less than 6 months and reducing for more than 6 months.

Parameter	No reduction of the sweets intake		Reduction of the sweets intake <6 months		Reduction of the sweets intake >6 months	
	Mean±SD	Median	Mean±SD	Median	Mean±SD	Median
FG scale	6.9±10	3	7.48±5.54	6.5	5.18±5.9	3
Acne	1.17±1.02	1	1.43±1.21	1	1.18±1.22	1
BMI (kg/m2)	26.08±6.43	24.34	27.3±6.1	26.46	23.58±5.2	23.3
WHR	0.81±0.08	0.79	0.81±0.07	0.83	0.79±0.08	0.77
TC (mg/dl) (<190)	171.57±35.62	161.5	177.17±33.6	173.5	171.75±30.24	167.5
HDL-C (mg/dl) (>40)	57.57±12.96	58.3	53.34±14.84	49.3	55.95±11.44	56.05
LDL-C (mg/dl) (<135)	96.16±24.02	92.5	102.38±30.13	97.7	97.39±30.7	94.15
TG (mg/dl) (<150)	100.46±56.76	86.1	107.3±50.4	98.6	89.36±57	77.05
TSH (µIU/ml) (0.27-4.2)	1.87±0.94	1.65	1.9±1.25	1.64	1.82±0.92	1.78
FT4 (ng/dl) (0.93-1.71)	1.21±0.16	1.19	1.24±0.16	1.25	1.23±0.11	1.23
Fasting Glucose (mg/dl) (70-99)	85.37±6.63	84.2	84.26±5.8	84.15	84.36±7.18	84.05
Glucose 120 min OGTT (mg/dl)(<140)	116.66±32.38	111	111.72±30.84	105.5	113.71±29.65	113
Fasting INS (µU/ml) (2.6-24.9)	9.8±6.95	7.4	9.54±5.46	8.44	7.45±4.37	5.69
HOMA-IR	2.12±1.63	1.54	2.01±1.2	1.71	1.57±0.95	1.23
SBP (mmHg) (<140)	124±12.21	125.5	126.31±11.25	126.5	123.96±10.11	120.5
DBP (mmHg) (<90)	78.51±9.51	79	78.52±9.28	79	78.89±9.09	77
SHBG (nmol/l) (32.4-128)	51.57±29.23	48.6	46.59±30.65	34.4	53.14±29.03	53.75
AMH (ng/ml) (1.2-9.05)	6.26±3.07	6.29	6.62±4.48	5	7.19±5.19	5.11
TT (ng/ml) (0.084-0.481)	0.681±1.9	0.4	0.43±0.21	0.37	0.359±0.18	0.335
TF (pg/ml) (0.1-6.3)	2.89±3.11	2.04	2.97±2.18	2.235	1.89±1.49	1.48
A4 (ng/ml) (0.49-1.31)	1.59±0.56	1.52	1.69±0.73	1.5	1.72±0.76	1.47

(FG – assessment of hirsutism on the Ferriman-Gallwey scoring system; BMI - body mass index; WHR - waist to hip ratio; TC - Total cholesterol, HDL-C -high-density lipoprotein cholesterol; LDL-C - low-density lipoprotein cholesterol; TG - triglycerides; TSH - thyroid-stimulating hormone; FT4 - free thyroxine; INS - insulin; HOMA-IR - homeostatic assessment of insulin resistance; SBP - Systolic blood pressure; DBP - Diastolic blood pressure; SHBG - sex hormone binding globulin ; AMH - anti-Mullerian hormone; TT - total testosterone; TF - free testosterone, A4 – androstendione)

Table 2. Parameters of patients divided into three groups: not reducing soft drinks in their diet, reducing for less than 6 months and reducing for more than 6 months.

Parameter	No reduction of the soft drinks intake		Reduction of the soft drinks intake <6 months		Reduction of the soft drinks intake >6 months	
	Mean±SD	Median	Mean±SD	Median	Mean±SD	Median
FG scale	5.22±5	3	7.96±6.28	8	5.72±6.86	3
Acne	1.22±1.18	1	1.44±1.18	1	1.15±1.06	1
BMI (kg/m2)	25.65±6.61	24	28.1±6.17	26.35	24.91±5.64	23.78
WHR	0.80±0.08	0.80	0.81±0.08	0.82	0.80±0.08	0.78
TC (mg/dl) (<190)	171.5±27.7	164.5	177.34±35.16	173.5	171.85±35.7	170
HDL-C (mg/dl) (>40)	56.35±10.4	56.1	51.33±12.1	50.2	58.85±14.86	58.8
LDL-C (mg/dl) (<135)	96.36±24.1	90.08	103.16±30.78	97.7	96.34±26.5	94.6
TG (mg/dl) (<150)	94±56.5	77.85	112.2±50.3	103	95.1±54.9	77.3
TSH (μIU/ml) (0.27-4.2)	1.89±1.06	1.62	2.01±1.24	1.67	1.76±0.89	1.69
FT4 (ng/dl) (0.93-1.71)	1.17±0.13	1.17	1.24±0.14	1.26	1.24±0.17	1.23
Fasting Glucose (mg/dl) (70-99)	85.25±4.88	85.05	84.38±7.1	83.65	84.89±6.63	84.1
Glucose 120 min OGTT (mg/dl)(<140)	110.12±23.75	107	115.62±30.67	112	115.43±34.64	108
Fasting INS (μU/ml) (2.6-24.9)	9.04±6.31	6.49	10.72±6.72	8.45	8.43±5.41	7.1
HOMA-IR	1.92±1.36	1.37	2.14±1.27	1.71	1.89±1.49	1.48
SBP (mmHg) (<140)	122.6±13.3	123	128.5±9.8	128	123.3±11.2	120
DBP (mmHg) (<90)	78±9.3	77	81.16±8.56	80	77.06±9.5	77.5
SHBG (nmol/l) (32.4-128)	49.9±22.3	5.65	40.24±23.52	36.3	57.02±34.46	53.7
AMH (ng/ml) (1.2-9.05)	6.47±2.97	6.34	6.63±4.98	5.04	6.54±3.72	5.55
TT (ng/ml) (0.084-0.481)	0.416±0.21	0.36	0.499±0.239	0.47	0.386±0.162	0.36
TF (pg/ml) (0.1-6.3)	2.35±2.28	1.65	3.61±3.36	3.04	2.36±2.01	1.93
A4 (ng/ml) (0.49-1.31)	1.547±0.55	1.49	1.74±0.72	1.51	1.64±0.66	1.5

(FG – assessment of hirsutism on the Ferriman-Gallwey scoring system; BMI - body mass index; WHR - waist to hip ratio; TC - Total cholesterol, HDL-C -high-density lipoprotein cholesterol; LDL-C - low-density lipoprotein cholesterol; TG - triglycerides; TSH - thyroid-stimulating hormone; FT4 - free thyroxine; INS - insulin; HOMA-IR - homeostatic assessment of insulin resistance; SBP - Systolic blood pressure; DBP - Diastolic blood pressure; SHBG - sex hormone binding globulin ; AMH - anti-Mullerian hormone; TT - total testosterone; TF - free testosterone, A4 – androstendione).

Table 3. Parameters of patients divided into three groups: not reducing red, fatty meat in their diet, reducing for less than 6 months and reducing for more than 6 months.

**Parameter**	**No reduction of the red, fatty meat**		**Reduction of the red, fatty meat intake <6 months**		**Reduction of the red, fatty meat intake >6 months**	
	Mean±SD	Median	Mean±SD	Median	Mean±SD	Median
FG scale	6.62±6.57	5.5	7.41±6.64	6	6.61±10.2	3
Acne	1±1.13	1	1.43±0.96	1	1.37±1.15	1
BMI (kg/m2)	27.4±7.2	25.5	25.88±5.28	24.4	25.29±5.64	23.78
WHR	0.82±0.08	0.83	0.79±0.07	0.78	0.79±0.08	0.78
TC (mg/dl) (<190)	171.57±36.99	173	174.72±33.45	171	174.44±32.26	169
HDL-C (mg/dl) (>40)	54.47±12.92	54.6	55.94±12.16	58.1	56.6±14.34	56.8
LDL-C (mg/dl) (<135)	98.5±27.2	92.95	99.54±29.14	97.7	98.23±27.44	92.5
TG (mg/dl) (<150)	109.25±62.8	93.45	96.28±40.85	90.9	95.99±53.35	79.1
TSH (μIU/ml) (0.27-4.2)	1.94±1.38	1.52	1.91±1	1.76	1.81±0.8	1.75
FT4 (ng/dl) (0.93-1.71)	1.2±0.16	1.18	1.23±0.14	1.23	1.23±0.15	1.24
Fasting Glucose (mg/dl) (70-99)	85.87±6.77	84.4	84.4±7.12	85.3	84.25±5.9	83.3
Glucose 120 min OGTT (mg/dl)(<140)	120.36±33.86	118	114.12±28.9	111	110.57±30.1	105
Fasting INS (μU/ml) (2.6-24.9)	10.87±7.65	7.82	8.92±5.08	7.01	8.38±5.04	7.08
HOMA-IR	2.36±1.8	1.72	1.88±1.1	1.58	1.77±1.13	1.47
SBP (mmHg) (<140)	125±12.15	125.5	121.72±10.3	123	125.85±11.4	125
DBP (mmHg) (<90)	78.48±9.63	77	77±9.89	78	79.3±8.9	79
SHBG (nmol/l) (32.4-128)	46.27±27.05	40.9	46.52±33.36	40.7	54.05±29.6	52.5
AMH (ng/ml) (1.2-9.05)	6±0.38	4.9	8±4.9	6.5	6.42±3.7	5.32
TT (ng/ml) (0.084-0.481)	0.787±2.19	0.39	0.499±0.22	0.51	0.375±0.18	0.34
TF (pg/ml) (0.1-6.3)	3.13±2.7	2.44	3.266±2.07	2.94	2.31±2.67	1.55
A4 (ng/ml) (0.49-1.31)	1.6±0.6	1.5	2.04±0.83	1.88	1.54±0.57	1.43

(FG – assessment of hirsutism on the Ferriman-Gallwey scoring system; BMI - body mass index; WHR - waist to hip ratio; TC - Total cholesterol, HDL-C -high-density lipoprotein cholesterol; LDL-C - low-density lipoprotein cholesterol; TG - triglycerides; TSH - thyroid-stimulating hormone; FT4 - free thyroxine; INS - insulin; HOMA-IR - homeostatic assessment of insulin resistance; SBP - Systolic blood pressure; DBP - Diastolic blood pressure; SHBG - sex hormone binding globulin ; AMH - anti-Mullerian hormone; TT - total testosterone; TF - free testosterone, A4 – androstendione).

Table 4. Parameters of patients divided into three groups: not reducing alcohol in their diet, reducing for less than 6 months and reducing for more than 6 months.

Parameter	No reduction of the alcohol intake		Reduction of the alcohol intake <6 months		Reduction of the alcohol intake >6 months	
	Mean±SD	Median	Mean±SD	Median	Mean±SD	Median
FG scale	7.58±13	3	8.6±6.8	6	6.49±6.72	5
Acne	1.08±1.2	1	1.38±1.06	1	1.29±1.11	1
BMI (kg/m2)	26.48±6.9	25.28	26.61±6.57	23.78	25.79±5.77	24.73
WHR	0.79±0.09	0.77	0.8±0.07	0.78	0.81±0.08	0.81
TC (mg/dl) (<190)	177.62±25.49	176	179.38±33.07	174	170.33±36.8	166
HDL-C (mg/dl) (>40)	57.38±12.8	58.15	54.31±12.9	53.75	55.58±13.9	55
LDL-C (mg/dl) (<135)	98.03±21.66	91.7	103.38±26.87	98.44	97.41±29.75	93.97
TG (mg/dl) (<150)	109.64±65.06	90.45	108.46±49.87	101.8	96.05±51.2	83.95
TSH (μIU/ml) (0.27-4.2)	1.94±0.99	1.62	1.92±1.24	1.695	1.83±1.02	1.66
FT4 (ng/dl) (0.93-1.71)	1.23±0.19	1.25	1.2±0.14	1.24	1.22±0.14	1.21
Fasting Glucose (mg/dl) (70-99)	84.94±6.26	84	83.92±6.16	83.4	84.99±6.63	84.6
Glucose 120 min OGTT (mg/dl)(<140)	116.16±26.8	113.5	112.81±30.44	108	114.13±33.31	106
Fasting INS (μU/ml) (2.6-24.9)	8.95±4.66	7.38	9.88±6.33	7.83	9.24±6.51	7.29
HOMA-IR	1.9±1.08	1.49	2.08±1.36	1.67	1.98±1.51	1.54
SBP (mmHg) (<140)	124.9±12.3	126	123.1±12.8	123	125.2±10.9	124.5
DBP (mmHg) (<90)	78±10.2	77	77.3±11.3	77.5	79.2±8.3	80
SHBG (nmol/l) (32.4-128)	50.49±31	43	52.85±35.97	41.85	49.19±27.3	45.85
AMH (ng/ml) (1.2-9.05)	5.86±3.48	4.88	7.51±4.87	5.77	6.56±3.94	5.64
TT (ng/ml) (0.084-0.481)	0.917±2.65	0.425	0.486±0.21	0.47	0.388±0.19	0.34
TF (pg/ml) (0.1-6.3)	3.04±2.5	2.19	3.54±3.43	2.77	2.384±2.32	1.63
A4 (ng/ml) (0.49-1.31)	1.64±0.58	1.5	1.83±0.74	1.75	1.61±0.66	1.43

(FG – assessment of hirsutism on the Ferriman-Gallwey scoring system; BMI - body mass index; WHR - waist to hip ratio; TC - Total cholesterol, HDL-C -high-density lipoprotein cholesterol; LDL-C - low-density lipoprotein cholesterol; TG - triglycerides; TSH - thyroid-stimulating hormone; FT4 - free thyroxine; INS - insulin; HOMA-IR - homeostatic assessment of insulin resistance; SBP - Systolic blood pressure; DBP - Diastolic blood pressure; SHBG - sex hormone binding globulin ; AMH - anti-Mullerian hormone; TT - total testosterone; TF - free testosterone, A4 – androstendione).
